# Recognition of *Porphyromonas gingivalis* Gingipain Epitopes by Natural IgM Binding to Malondialdehyde Modified Low-Density Lipoprotein

**DOI:** 10.1371/journal.pone.0034910

**Published:** 2012-04-05

**Authors:** S. Pauliina Turunen, Outi Kummu, Kirsi Harila, Marja Veneskoski, Rabah Soliymani, Marc Baumann, Pirkko J. Pussinen, Sohvi Hörkkö

**Affiliations:** 1 Department of Medical Microbiology and Immunology, Institute of Diagnostics, University of Oulu, Oulu, Finland; 2 Clinical Research Center, Oulu University Hospital, Oulu, Finland; 3 Protein Chemistry Unit, Institute of Biomedicine/Anatomy, Biomedicum Helsinki, Helsinki, Finland; 4 Institute of Dentistry, University of Helsinki, Helsinki, Finland; Universität Würzburg, Germany

## Abstract

**Objective:**

Increased risk for atherosclerosis is associated with infectious diseases including periodontitis. Natural IgM antibodies recognize pathogen-associated molecular patterns on bacteria, and oxidized lipid and protein epitopes on low-density lipoprotein (LDL) and apoptotic cells. We aimed to identify epitopes on periodontal pathogen *Porphyromonas gingivalis* recognized by natural IgM binding to malondialdehyde (MDA) modified LDL.

**Methods and Results:**

Mouse monoclonal IgM (MDmAb) specific for MDA-LDL recognized epitopes on *P. gingivalis* on flow cytometry and chemiluminescence immunoassays. Immunization of C57BL/6 mice with *P. gingivalis* induced IgM, but not IgG, immune response to MDA-LDL and apoptotic cells. Immunization of LDLR^−/−^ mice with *P. gingivalis* induced IgM, but not IgG, immune response to MDA-LDL and diminished aortic lipid deposition. On Western blot MDmAb bound to *P. gingivalis* fragments identified as arginine-specific gingipain (Rgp) by mass spectrometry. Recombinant domains of Rgp produced in *E. coli* were devoid of phosphocholine epitopes but contained epitopes recognized by MDmAb and human serum IgM. Serum IgM levels to *P. gingivalis* were associated with anti-MDA-LDL levels in humans.

**Conclusion:**

Gingipain of *P. gingivalis* is recognized by natural IgM and shares molecular identity with epitopes on MDA-LDL. These findings suggest a role for natural antibodies in the pathogenesis of two related inflammatory diseases, atherosclerosis and periodontitis.

## Introduction

Atherosclerosis is a chronic inflammatory disease [1] and infections potentially contribute to atherogenesis with local inflammatory changes on the vascular wall [2]. Conventional risk factors of cardiovascular disease (CVD) such as hypercholesterolemia, hypertension, smoking or diabetes can be attributed to the majority of CVD incidences but the effects of genetic and environmental factors are also acknowledged [3]. Viral and bacterial pathogens are encountered by the immune system, and many studies report that infectious burden with several pathogens associates with the progression of atherosclerosis independently of classic risk factors [4].

Lipoprotein oxidation is essential for initiation and progression of atherosclerosis. Oxidized lipid epitopes are generated on low-density lipoprotein (LDL) by lipid peroxidation reactions of polyunsaturated fatty acids producing reactive aldehydes, such as malondialdehyde (MDA) and 4-hydroxynonenal, which form covalent adducts with lysine residues of apolipoprotein B or amino-containing phospholipids of LDL [5], [6]. Additionally, oxidation of phospholipids exposes the phosphocholine (PC) moiety, which is found on oxidized LDL (OxLDL) and bacterial cell wall polysaccharides on for example *Streptococcus pneumoniae* [7]. Oxidatively modified lipoproteins participate in the inflammatory processes of atherogenesis and contain highly immunogenic epitopes that are targeted by the natural antibodies of the innate immune system [8].

Infectious pathogens and apoptotic cells have also been reported to contain structural motifs recognized by natural antibodies [9]. Oxidative modifications of lipoproteins generate epitopes that are a class of pathogen-associated molecular patterns (PAMP) or danger-associated molecular patterns (DAMP) recognized by components of innate immune system [10]. Natural IgM antibodies recognize the molecular mimicry between epitopes of bacterial PAMPs and OxLDL [9], and autoantibodies to oxidized lipid and protein epitopes on LDL are present in both humans and mice [11]–[13]. IgM antibodies to OxLDL are reported to be required for atheroprotection in mice [14] although some controversial data [15] exists. LDL receptor (LDLR^−/−^) and apolipoprotein E –deficient (apoE^−/−^) mice generate autoantibodies to MDA modified LDL (MDA-LDL) representing epitopes different from the structure of PC, and immunization of mice with MDA-LDL has been demonstrated to reduce atheroprogression [11], [16]. MDA and related aldehydes are abundantly generated in biological lipid peroxidation [17], and already at the time of birth humans have natural IgM recognizing MDA epitopes [18]. Epidemiological studies in humans suggest that IgM antibodies to OxLDL are associated with lower incidence of atherosclerosis [12]. IgG antibodies to OxLDL, on the contrary, have been reported to associate with an increased risk for atherosclerosis [13]. IgM antibodies to oxidized lipid and protein epitopes are proposed to reduce foam cell formation by blocking the uptake of OxLDL by macrophage scavenger receptors [19], [20].

Periodontal diseases cause a progressive destruction of the tooth-supporting tissue and are associated with an increased risk for CVD in humans [21], [22]. *Porphyromonas gingivalis* is considered as one of the principal pathogens for chronic adult periodontitis. In animal models, infection with live *P. gingivalis* accelerates atherosclerosis in homo- and heterozygous apoE-deficient mice [23]–[27]. Conversely, atheroprogression can be prevented with *P. gingivalis* immunization using heat-killed bacteria [25], [28]. The major virulence factors of *P. gingivalis* include fimbriae, lipopolysaccharides and various proteases and hemagglutinins. Proteases named arg-gingipain (Rgp) and lys-gingipain (Kgp) are important for the virulence of *P. gingivalis* as they elicit dysfunction of inflammatory and immune responses and can degrade various connective tissue proteins [29]. However, precise molecular mechanisms of how oral pathogens accelerate atherosclerosis are still unclear.

The aim of the present study was to characterize new epitope cross-reactivity, other than the previously described PC, between oxidized LDL and bacteria. We focused on natural IgM against MDA epitopes on LDL because MDA is a naturally occurring aldehyde targeted by antibodies present at birth and IgM antibodies to MDA-LDL associate with diminished atheroprogression. We screened few bacterial species for recognition by anti-MDA-LDL IgM, and *P. gingivalis* was assessed in more detail since some strains of *P. gingivalis* are previously determined as devoid of PC [30]. We investigated the effect of *P. gingivalis* immunization on the induction of anti-MDA-LDL antibodies and on the progression of atherosclerosis in mice. Further, we identified proteins of *P. gingivalis* recognized by anti-MDA-LDL-IgM with mass spectrometry, and assessed if human sera IgM recognized the *P. gingivalis* epitope sharing molecular identity to MDA-LDL.

## Methods

### Cloning of mouse monoclonal IgM

Splenocytes from a naïve apoE^−/−^ mouse (C57BL/6 background B6.129P2-*Apoe^tm1^*N11, Taconic, Denmark) were fused with P3×63Ag8.653.1 myeloma cells using standard methods. Splenocyte-myeloma hybridoma cells were grown in Dulbecco's modified Eagle's medium (Sigma, St. Louis, MO) supplemented with 20% fetal bovine serum (HyClone laboratories, South Logan, UT, USA), 1% non-essential amino acids, 10 mM HEPES, 50 µM β-mercaptoethanol, 100 U/ml penicillin and 100 µg/ml streptomycin (Sigma, St. Louis, MO, USA). Cells were maintained in a humidified atmosphere with 5% carbon dioxide at +37°C. For large scale production of mouse mAbs the hybridomas were cultured in serum-free HyClone SFM4MAb-Utility medium (HyClone Laboratories) with antibiotics for 12 days. Hybridoma cultures were tested for production of IgM to MDA-LDL, and IgM was purified with Sepharose 6 100/300 GL gel filtration column (GE Healthcare Bio-Sciences, Piscataway, NJ, USA) and analyzed for purity on sodiumdodecylsulphate polyacrylamide gel electrophoresis (SDS-PAGE). Mouse monoclonal IgM, MDmAb (clone HME-04_7), was selected for this study. According to the sequence analysis the variable regions of MDmAb's heavy and kappa light chain were >90% identical to the corresponding mouse germ-line gene sequences (data not shown). Isotype control IgM (clone HMC-06_29) was generated from a naïve C57BL/6 mouse using similar methods. Anti-PC-IgM control antibody (EO6) [31] was a kind gift from Professor J. Witztum, University of California, San Diego, USA.

### LDL isolation and modifications

Low-density lipoprotein fraction (density 1.019–1.063 g/ml) was isolated from human plasma by sequential density gradient centrifugation [32]. Malondialdehyde (MDA) modification to LDL was prepared as described [33]. In brief, 0.5 M solution of 1,1,3,3 – tetramethoxypropane malonaldehyde-bis(dimethyl acetal) (Sigma Aldrich) in 0.3% hydrochloric acid was incubated at +37°C for 10 minutes. pH was adjusted between 6.0 and 7.0 with sodium hydroxide and the final volume was adjusted to 4 ml with sterile water. A total of 900 µl of 0.5 M MDA was added to 6 mg LDL and the volume was adjusted to 3 ml. The mixture was incubated at +37°C for three hours and dialysed extensively against 0.27 mM EDTA (ethylenediaminetetraacetic acid) in phosphate buffered saline (PBS). The percentage of modified lysine residues was determined with TNBS (2,4,6 – trinitrobenzene sulphonic acid trihydrate; Fluka Chemika) testing [34]. Malondialdehyde acetaldehyde (MAA) modification was performed as described [35], [36]. 0.5 M MDA solution was prepared as described and pH was adjusted to 4.8. A total volume of 310 µl PBS, 140 µl of 20% acetaldehyde, 5 mg LDL and 300 µl 0.5 M MDA were mixed in this order. pH was adjusted to 4.8 and the mixture was incubated at +37°C for two hours. Buffer of MAA-LDL was changed into 0.27 mM EDTA in PBS with Amicon Ultra-4 Ultracel MWCO 10,000 filters (Millipore). Fluorescent MAA-residues were measured at excitation wavelength of 355 nm and emission wavelength of 460 nm with Victor^2^ multilabel reader (Perkin Elmer). Copper oxidized LDL (CuOx-LDL) was prepared by incubating LDL with 4 mM CuSO_4_ for 24 hours at +37°C. Reaction was stopped by adding EDTA to a final concentration of 200 µM, and LDL was dialysed [37].

### Bacteria

Oral pathogenic bacteria *Porphyromonas gingivalis* (Pg) strains representing three serotypes, ATCC 33277, W50 and OMGS 434, were used in the study and cultured on *Brucella* agar plates supplemented with 5% horse blood, 5 µg/ml hemin and 100 mg/ml vitamin K_1_ anaerobically for 5 to 6 days. Purity of the cultures was checked by colony morphology and Gram-staining. Heat-killed *P. gingivalis* was prepared by incubation at 60°C for one hour in PBS, and used for immunizing C57BL/6 mice. For other assays, including immunization of LDLR^−/−^ mice, the bacteria were fixed overnight in 0.5% formalin in PBS and washed extensively with PBS. *P. gingivalis* suspensions were adjusted to an absorbance of 0.15 at 580 nm for chemiluminescence immunoassays and three strains were mixed in equal volumes [38]. For flow cytometry assays *P. gingivalis* cells were picked from plates and suspended in PBS. *Escherichia coli* BL21(DE3) (Invitrogen, Eugene, OR, USA) cells were washed from culture medium with PBS for flow cytometry and treated with 0.5% formalin in PBS overnight for chemiluminescence immunoassay.

### Chemiluminescence immunoassays and Western blotting

MDA-LDL, MAA-LDL, native LDL and PC-conjugated bovine serum albumin (PC-BSA) (Biosearch Technologies, Novato, CA, USA) were used as antigens (5 µg/ml) in chemiluminescence immunoassays [12], [33]. *P. gingivalis* suspensions were adjusted to an absorbance of 0.15 at 580 nm and three strains were mixed in equal volumes. Specific binding of MDmAb to bacteria, modified and native LDL, PC-BSA and recombinant domains of gingipain (Rgp) was tested with competitive chemiluminescence immunoassay. MDA- and MAA-modified, native LDL and PC-BSA (0–100 µg/ml) and Rgps (0–200 µg/ml) were incubated with 0.3 µg/ml MDmAb diluted in 0.5% fish gelatin – 0.27 mM EDTA in PBS (FG-buffer) overnight at +4°C and the samples were centrifuged at 16,000× g at +4°C. 96-well microtiter plates with 5 µg/ml immobilized antigens were washed with 0.27 mM EDTA in PBS three times between each step of the immunoassay. FG-buffer was used for blocking the plates before adding 1.25 µg/ml mouse monoclonal antibodies and diluted plasma in duplicates or competition samples in triplicates. Alkaline phosphatase conjugated secondary antibodies (Sigma, St. Louis, MO, USA) and Lumiphos 530 substrate (Lumigen Inc. Southfield, MI, USA) were used for chemiluminescence detection. IgM binding to bacteria and recombinant proteins was analyzed by SDS-PAGE and Western blotting using anti-mouse-IgM-Alexa Fluor 680 antibody (Invitrogen) for detection, and the nitrocellulose membranes were imaged with Odyssey infrared scanner (LI-COR, Lincoln, NE, USA).

### Protein identification by mass spectrometry and proteome data analysis

Excised gel bands matching to MDmAb immunostaining were washed and dehydrated with acetonitrile (ACN). Proteins were reduced with 20 mM dithiothreitol and incubated at 56°C for 30 min before alkylation with 55 mM iodoacetamide - 0.1 M ammonium hydrogen carbonate (NH_4_HCO_3_) in the dark at room temperature for 15 minutes. After washing with 0.1 M NH_4_HCO_3_ and dehydration with ACN the gel pieces were rehydrated in 10 to 15 µl sequencing grade modified trypsin (Promega, USA) in 0.1 M NH_4_HCO_3_, to a final concentration of 0.01 µg/µl trypsin and incubated for digestion overnight at 37°C. Tryptic peptides were eluted from the gel pieces by incubating successively in 25 mM NH_4_HCO_3_ and then twice in 5% formic acid for 15 minutes at room temperature each. The resulting tryptic digest peptides were desalted using Zip Tip µC-18 reverse phase columns (Millipore, USA) and directly eluted with 50% ACN - 0.1% trifluoroacetic acid (TFA) onto MALDI target plate. A saturated matrix solution of α-cyano-4-hydroxy cinnamic acid (CHCA) (Sigma, USA) in 33% ACN - 0.1% TFA was added [39].

MALDI-TOF analyses were carried out with Autoflex III (Bruker Daltonics, Bremen Germany) equipped with a SmartBeam™ laser (355 nm), operated in positive and reflective modes. Typically, mass spectra were acquired by accumulating spectra of 2000 laser shots and up to 10000 for MS/MS spectra. External calibration was performed for molecular assignments using a peptide calibration standard (Bruker Daltonik GmbH, Leipzig, Germany). Trypsin autolytic peptide masses were used to check or correct the calibration. These autolytic peptides and with keratin – derived ones, when present, were removed before search submission. Protein identifications were performed by combining the files (PMF and few Lift spectra (MSMS) originating from the same spot) and searching against SwissProt database. ‘Other bacteria’ was selected in taxonomy field (over 42100 sequences) using Matrix Science's Mascot (Matrix Science Ltd, UK). FlexAnalysis™ v3.0 and Biotools™ v3.1 softwares (Bruker Daltonics) were used to assign molecular isotopic masses to the peaks in the MS spectra and as search engine interface between mass list data transfer and the databases in Mascot server, respectively. The following parameters were set for the searches: 0.1 Da precursor tolerance and 0.5 or 1 Da MS/MS fragment tolerance for combined MS and MS/MS searches, fixed and variable modifications were considered (carbamidomethylated cysteine and oxidized methionine, respectively), one trypsin missed cleavage was allowed. Protein identifications were further evaluated by comparing the calculated and observed molecular masses, as well as the quality of MS/MS mass spectra and their amino acid sequence matching to the identified peptides [40].

### Production of recombinant gingipain

Purified genomic DNA of *P. gingivalis* ATCC 33277 was used as the template for amplifying *rgpA*. The primers were designed to amplify sequences coding for the catalytic domain (CAT) (amino acid residues 225–716) and two sequences at adhesin/hemagglutinin domain, Rgp44 (amino acid residues 717–1135) and Rgp15–27 (amino acid residues 1136–1703) (modified from Inagaki *et al.* 2003) [41]. The primer sequences for RgpCAT were forward 5′-GGTATTGAGGGTCGCATGTACACACCGGTAGAGGAAAAACAAAATGGTC-3′, and reverse 5′-AGAGGAGAGTTAGAGCCTTAGCGAAGAAGTTCGGGGGCATCGCTG-3′. For Rgp44 the forward primer was 5′-GGTATTGAGGGTCGCATGAGCGGTCAGGCCGAGATTGTTCTTG-3′ and the reverse 5′-AGAGGAGAGTTAGAGCCTTAGCGCTTGCCGTTGGCCTTGATCTC-3′. For Rgp15–27 the forward primer was 5′-GGTATTGAGGGTCGCATGGCAGACTTCACGGAAACGTTCGAGTC-3′ and the reverse 5′-AGAGGAGAGTTAGAGCCTTACTTTACAGCGAGTTTCTCTACGTAAG-3′. The polymerase chain reaction amplification was performed using the following cycles 35 times: denaturation at 98°C for 10 seconds, annealing and extension at 72°C for 30 seconds. PCR products were visualized on 1% agarose gel. Ligation of the inserts to the expression vector was performed by using pET32 Xa LIC vector kit according to manufacturer's instructions (Novagen, Madison, WI, USA). Purification of the histidine-tagged recombinant proteins followed the protocols of ProBond Purification System (Invitrogen, Carlsbad, CA, USA) with slight modifications. *E. coli* BL21(DE3) were harvested by centrifugation at 3,000× g for 10 minutes and suspended in native lysis buffer containing 50 mM Tris – 25 mM sodium chloride – 2.5 mM magnesium chloride – 0.1 mM calcium chloride pH 8.0. Lysozyme was added 1 mg/ml and incubated at 30°C for 30 minutes. Bacteria were sonicated 7×10 seconds and treated with DNase I (Fermentas) 4 U/ml for 15 minutes on ice. Cellular debris was collected by centrifugation at 3,000× g for 15 minutes. Soluble Rgp15–27 protein was purified from the supernatant. Recombinant Rgp44 and RgpCAT were expressed as inclusion bodies remaining in the cell pellet after native lysis. Rgp44 and -CAT were purified from *E. coli* cells by denaturing lysis with 6 M guanidium hydrochloride for 10 minutes. The lysate was homogenized with a needle and a syringe and sonicated 3×5 seconds. Viscous cell debris was separated by ultracentrifugation at 100,000× g for 30 minutes. Soluble proteins in the supernatant were bound to nickel chelating slurry following manufacturer's protocols. Phosphate buffers without urea were used in wash and elution steps for all Rgps. Purity of the histidine-tagged recombinant proteins was analyzed on SDS-PAGE. Small molecular weight protein contaminants were removed with Amicon Ultra-4 Ultracel MWCO 30,000 filters (Millipore) for immunoassays using Rgps as immobilized antigens. For detailed description of the purification protocol see Methods S1.

### Animals, diets and immunization

Female LDLR^−/−^ mice (C57BL/6J background B6.129S7-*Ldlr^tm1Her^*) were evenly grouped according to the age and weight and kept in conventional housing. Primary immunizations without adjuvant were performed for LDLR^−/−^ mice (n = 7) by subcutaneous injections of killed whole bacteria (50 µg protein) representing the three strains of *P. gingivalis* mixed in equal volumes [38]. Control group received sterile PBS (n = 8). Three boosters (25 µg protein) were given intraperitoneally at two-week intervals. During the immunization period LDLR^−/−^ mice were fed regular chow (4.4% fat and 0.01% cholesterol) followed by Western type high fat diet (HFD) (22% fat and 0.15% cholesterol (TD88137 Harlan Teklad, Madison, WI, USA) for 15–18 weeks. Booster injections were given once a month during HFD.

Female C57BL/6 mice on chow diet (n = 8) were immunized without adjuvant with one strain of heat-killed *P. gingivalis* (ATCC 33277, 2×10^8^ CFU) in sterile saline injected subcutaneously. We confirmed that the recognition of epitopes on *P. gingivalis* by MDmAb was not dependent on the strain i.e. gingipain was detected in immunoblots in all three strains similarly. Boosters (1×10^8^ CFU) were injected intraperitoneally three times every two weeks. Controls received sterile saline (n = 8).

Mouse blood samples before and during the immunizations were taken from the hind leg vein into Microvette CB 300 lithium-heparin tubes (Sarstedt, Nümbrecht, Germany) and plasma was separated at 2,000× g for 5 minutes. The mice were sacrificed with carbon dioxide inhalation and blood samples were immediately collected from the vena cava into syringes primed with EDTA and plasma was separated.

### Analysis of atherosclerosis and plasma lipids in mice

The LDLR^−/−^ mice were sacrificed by carbon dioxide inhalation and blood was collected from vena cava. The aorta was perfused for 10 minutes with PBS containing 1 µg/ml butylated hydroxytoluene (BHT) via a cannula inserted into the left ventricule and fixed by perfusion with formalin-sucrose solution pH 7.4 (10% formalin - 5% sucrose - 3 µM EDTA - 20 µM BHT) for 10 minutes. Dissected aortas were stained with Sudan IV (Sigma Aldrich, St. Louis, MO, USA). Each aorta was then longitudinally opened and pinned to obtain a flat preparation for *en face* lesion size analysis [42], [43]. Aortic plaque areas were determined with MCID Core 7.0 Image analysis software (InterFocus Imaging, Cambridge, UK). The extent of atherosclerosis at the aortic origin was determined with the same image analysis system. Aortic sections were collected throughout the length of the aortic valve leaflets. Three 5 µm thick sections were consecutively collected onto an object glass, and every third glass was stained with hematoxylin-eosin. All stained sections were analyzed for total plaque area. Lining of the plaque area followed adventitia-media border and intima-lumen border at the luminal edge. Cross-sectional area of the aorta was determined following the adventitia-media border [42], [43]. The final value of plaque area for each mouse was the mean of nine consecutive stained sections from the thickest region of the plaque. Plasma total cholesterol, total triglycerides and HDL cholesterol in LDLR^−/−^ mouse plasma were determined after HFD by enzymatic methods using commercial kits from Roche Diagnostics, Mannheim, Germany. LDL cholesterol was estimated using the formula of Friedewald [44].

### Flow cytometry for IgM binding to bacteria and apoptotic cells

Apoptosis was induced in human Jurkat T cells by UV-irradiation (51 mJ/cm^2^) [33], [45]. Apoptotic cells were identified with propidium iodide (PI) staining (1 µg/ml). Binding of C57BL/6 mouse plasma IgM to apoptotic cells, and mouse monoclonal IgM binding to bacteria was analyzed with FACSCalibur (BD Biosciences, San Jose, CA, USA) using anti-mouse-IgM Alexa Fluor 488 (Invitrogen). Data was analyzed using FCS Express V3 software (De Novo Software, Los Angeles, CA, USA).

### Human serum immunoassay

Fasting blood samples were collected from 29 healthy adult volunteers (5 men and 24 women) and serum was separated. Serum IgM and IgG binding to *P. gingivalis* and MDA-LDL was analyzed by chemiluminescence immunoassays. Competitive immunoassay was used for determining the specificity of human serum IgM binding to the recombinant gingipain.

### Ethics statement

The animal protocol was approved by the National Animal Experiment Board, Finland (STH1164A), and the animal studies were carried out according to the approved guidelines.

Collection of human serum samples was carried out in accordance with the Declaration of Helsinki and approved by the Ethical Committee of the Oulu University Hospital, Finland (21/2006). A written informed consent was obtained from each participant.

### Statistical analysis

Two-tailed Student's t-test was used for analyzing the significance of the differences between variables. P-values less than 0.05 were considered significant. * P<0.05, ** P<0.01; ns, non-significant. Data are presented as mean ± SD. Spearman rank correlation tests were performed to determine the relationship between the variables that had skewed distribution. The box-and-whisker plots represent quartiles, the median and the mean (black square). The whiskers denote the 10^th^ and 90^th^ percentiles and asterisks denote minimum and maximum values.

## Results

### Cross-reactive epitopes on OxLDL and *P. gingivalis* recognized by natural IgM

**Figure 1:**
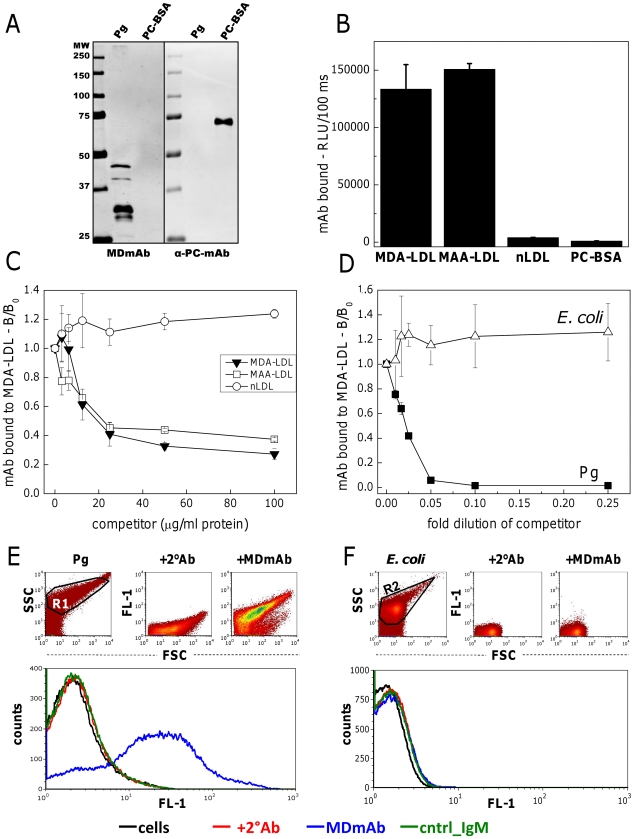
Cross-reactive epitopes on MDA-LDL and bacteria. Binding of anti-MDA-LDL-IgM (MDmAb) and anti-PC-IgM control antibody (α-PC-mAb) to *P. gingivalis* (Pg) and PC-conjugated bovine serum albumin (PC-BSA) on Western blot (A). Binding of MDmAb to MDA- and MAA-modified and native LDL (nLDL) and PC-BSA using direct binding (B) and competitive (C) chemiluminescence immunoassays. Specific binding of MDmAb to *P. gingivalis* (Pg) and *E. coli* was tested with competitive chemiluminescence immunoassay (D). Bacterial suspensions were adjusted to an absorbance of 0.15 at 580 nm with PBS and further diluted as indicated. B/B_0_ indicates the ratio of IgM binding with and without a competitor. RLU, relative light unit. Binding of MDmAb and isotype control (cntrl_IgM) to *P. gingivalis* (E) and *E. coli* (F) in native conditions was tested with flow cytometry. Fluorescence (FL-1) of the cells with the secondary antibody, +2°Ab (red), MDmAb (blue) and isotype control (green).

Mouse monoclonal IgM to MDA-LDL (MDmAb, clone HME-04_7) was analyzed for binding to bacterial epitopes on *P. gingivalis* (Pg). Western blotting was performed to assess binding of MDmAb to proteins of Pg (Fig. 1A). Four bacterial protein fragments of approximately 45, 40, 32 and 30 kDa were recognized by MDmAb. No binding to PC-BSA was observed. Anti-PC-IgM control antibody (α-PC-mAb) did not bind to Pg in Western blot (Fig. 1A) validating that the bacterial PC-epitopes were not present in the ATCC33277 strain. Direct binding immunoassay showed MDmAb binding to epitopes on MDA- and malondialdehyde acetaldehyde (MAA) modified LDL, but not to native LDL (nLDL) or PC-BSA (Fig. 1B). MDmAb bound specifically to MDA-LDL, MAA-LDL (Fig. 1C) and *P. gingivalis* (Fig. 1D) but not to nLDL (Fig. 1C) or *E. coli* (Fig. 1D) in competitive immunoassays. Binding of MDmAb to bacteria was further confirmed in native conditions by flow cytometry. MDmAb bound to *P. gingivalis* cells (Fig. 1E), but not to *E. coli* (Fig. 1F). Thus, recognition of *P. gingivalis* by natural IgM was demonstrated to occur through epitopes sharing molecular mimicry with MDA-LDL.

### Epitopes for MDmAb in *P. gingivalis* are gingipain protease domains

**Figure 2:**
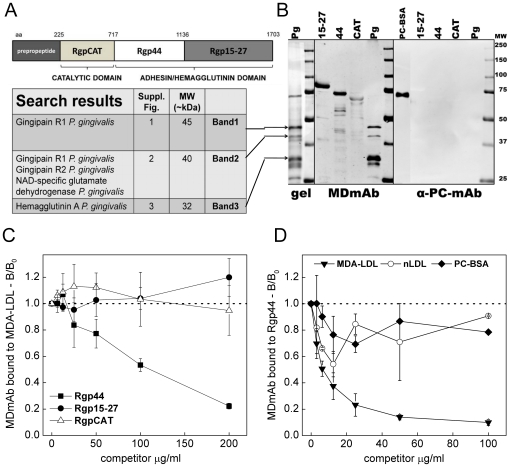
Identification of *P. gingivalis* epitopes for anti-MDA-LDL-IgM. A) Schematic presentation of arg-gingipain (RgpA) functional domains cloned and produced in a recombinant system [41]. B) Proteins of *P. gingivalis* were separated on SDS-PAGE (Pg, gel). Fragments recognized by MDmAb (45, 40 and 32 kDa, black arrows) were identified by mass spectrometry as arginine-specific gingipain or hemagglutinin A of *P. gingivalis*. Figures S1, S2, S3 contain the Mascot results of the database searches and the MSMS spectrum showing the matching amino acids in the peptide sequence. Three domains of the recombinant arg-specific gingipain, RgpCAT, Rgp44 and Rgp15–27 were produced in *E. coli* and analyzed for recognition by MDmAb and anti-PC-IgM control antibody (α-PC-mAb). C) Specific binding of MDmAb to recombinant gingipain domains Rgp15–27, Rgp44 and RgpCAT was tested with a competitive immunoassay. D) Reciprocally, soluble MDA-LDL, nLDL and PC-BSA were used as competitors for MDmAb binding to Rgp44. B/B_0_ indicates the ratio of IgM binding with and without a competitor. MW, molecular weight. PC-BSA, phosphocholine-conjugated bovine serum albumin.

Proteins in *P. gingivalis* containing epitopes for MDmAb were in-gel trypsin digested and identified by MALDI-TOF mass spectrometry (MS). Three protein fragments corresponding to MDmAb immunostaining (45 kDa Band1, 40 kDa Band2 and 32 kDa Band3) were analyzed in repeated MS assays, and frequently the highest match scores were obtained for gingipains or hemagglutinin. Arginine-specific gingipain (Rgp) was the most prevalent result in every MS analysis (see Figures S1, S2, and S3). Based on this data the recombinant domains of Rgp were produced in *E. coli* (Fig. 2A). Catalytic domain (RgpCAT) and two peptides in adhesin/hemagglutinin domain, Rgp44 and Rgp15–27, were analyzed for recognition by MDmAb (Fig. 2B). MDmAb showed strong binding to Rgp44 and Rgp15–27 and only weak binding to the catalytic domain on Western blot. Anti-PC IgM control antibody (α-PC-mAb) did not recognize epitopes on recombinant gingipain domains (Fig. 2B). Competitive immunoassay of MDmAb binding to Rgp domains showed specific binding to Rgp44 but not to Rgp15–27 or RgpCAT (Fig. 2C). MDA-LDL was an effective competitor for MDmAb binding to Rgp44, whereas native LDL and PC-BSA did not compete for MDmAb binding to Rgp44 (Fig. 2D). The molecular weights of the recombinant Rgps and the gingipain of *P. gingivalis* cell extract are very different on SDS-PAGE. Purified gingipain from *P. gingivalis* cells has been documented to resolve on SDS-PAGE under denaturing and reducing conditions to five bands of 45 kDa or less [46]. Similarly, small protein bands of 30, 27 and 18 kDa of arg-gingipain have been reported after boiling treatment [47]. In addition to conditions of SDS-PAGE the other possible explanation for observing gingipain in bands less than 45 kDa in *P. gingivalis* lysate is the autoproteolytic activity of the gingipain protease itself [48].

### IgM titers to MDA-LDL are elevated in C57BL/6 and LDLR^−/−^ mice after immunization with *P. gingivalis*

**Figure 3:**
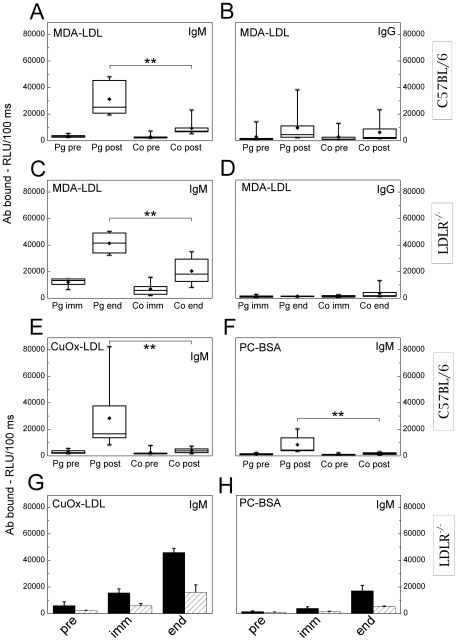
Mouse plasma IgM and IgG binding to MDA-LDL after immunization with *P. gingivalis*. C57BL/6 mice were immunized with heat-killed *P. gingivalis* ATCC33277 (Pg; n = 8) and controls received saline (Co; n = 8). Plasma IgM (A) and IgG (B) to MDA-LDL before (pre) and after immunization (post) were determined with chemiluminescence immunoassay. Each C57BL/6 plasma sample (1∶500) was measured in duplicate and an average for each individual was calculated. LDLR^−/−^ mice were immunized with killed *P. gingivalis* (3 strains mixed) (Pg; n = 7) and controls received PBS (Co; n = 8). Plasma IgM (C) and IgG (D) to MDA-LDL after the second booster immunization (imm) and after the HFD (end) were determined. Each LDLR^−/−^ plasma sample (1∶1000) was measured in duplicate and an average for each individual in two repeated assays was calculated. Additionally, mouse plasma IgM binding to CuOx-LDL (E, G) and PC-BSA (F, H) was determined. For C57BL/6 mice (E, F) this was done similarly as described for panel A. Plasma samples of LDLR^−/−^ mice (G, H) were pooled between three or four mice (1∶1000) for a single assay, in which the mean ± SD within a group is shown.

C57BL/6 mice immunized with heat-killed *P. gingivalis* showed a significant increase in plasma IgM binding to MDA-LDL compared to the controls (Fig. 3A). High fat fed LDLR^−/−^ mice immunized with killed *P. gingivalis* had also significantly increased plasma IgM binding to MDA-LDL compared to controls (Fig. 3C). Levels of IgM and IgG against MDA-LDL before immunization of LDLR^−/−^ mice are shown in the Figure S5. Pg-immunization did not significantly elevate IgG antibodies to MDA-LDL in C57BL/6 (Fig. 3B) or LDLR^−/−^ mice (Fig. 3D) compared to the controls. C57BL/6 and LDLR^−/−^ mice immunized with *P. gingivalis* had elevated titers to Pg-antigens as shown in Table 1. Plasma IgM binding to CuOx-LDL was also increased in *P. gingivalis* immunized C57BL/6 (Fig. 3E) and LDLR^−/−^ (Fig. 3G) mice, which was most likely due to the presence of MDA epitopes in CuOx-LDL as previously shown with other MDA-LDL specific monoclonal antibodies [11]. Low levels of plasma IgM binding to PC epitope in BSA carrier (PC-BSA) were observed in the Pg-immunized C57BL/6 mice (Fig. 3F) whereas the Pg-immunized LDLR^−/−^ mice had no increase in plasma IgM binding to PC-BSA (Fig. 3H). IgM binding to oxidized cardiolipin was not significantly elevated in C57BL/6 mice immunized with *P. gingivalis* compared to control mice (Fig. S4). Plasma lipids were analyzed in the LDLR^−/−^ mice at the end of HFD (Table 2). No significant differences were observed in the plasma total cholesterol or triglycerides, or LDL and HDL cholesterol levels between the groups.

**Table 1:** Plasma antibody levels to *P. gingivalis* in immunized mice.

Antibodies to Pg (RLU/100 ms)		C57BL/6*		LDLR^−/−^ †		
Group		pre	post	pre	immunized	after HFD
Pg	IgM	339±182	1529±750	202±22	1988±1293	10917±2642
Pg	IgG	274±62	21284±7410	175±76	5805±4130	6761±6558
controls	IgM	291±77	372±107	231±132	219±43	423±121
controls	IgG	317±77	331±92	191±42	267±95	235±53

* C57BL/6 female mice (n = 8 per group) were immunized with heat-killed *P. gingivalis* (Pg) (ATCC33277) and controls received saline. Plasma IgM and IgG to Pg before (pre) and after immunization (post) were determined with chemiluminescence immunoassay. Values are mean ± SD. RLU, relative light units.

† LDLR^−/−^ mice were immunized with killed *P. gingivalis* (n = 7 in Pg-immunized and n = 8 in PBS-controls) and plasma IgM and IgG to Pg before (pre), after the second booster immunization (immunized) and after the high fat diet (HFD) were determined. Values are mean ± SD. RLU, relative light units.

**Table 2:** Weight gain and plasma lipids of LDLR^−/−^ mice.

	Pg-immunized*	controls*
weight at start point of HFD (g)	28.4±2.5	30.5±4.2
Increase in weight during HFD (%)	6.3±4.7	7.4±5.0
HDL cholesterol (mmol/l)†	12.4±3.7	13.2±4.3
LDL cholesterol (mmol/l)†	25.5±9.2	30.3±9.1
total plasma cholesterol (mmol/l)†	40±13	47±15
total plasma triglycerides (mmol/l)†	6.4±2.6	7.6±3.6

* Female LDLR^−/−^ mice were immunized with killed *P. gingivalis* (n = 7) and controls (n = 8) received PBS. Mean ± SD is shown.

† Plasma lipids were measured from EDTA-plasma samples collected immediately after sacrifice at the end of HFD. Concentration of LDL cholesterol was estimated using the formula of Friedewald.

### Antibody responses to recombinant gingipain in *P. gingivalis* immunized mice

**Figure 4:**
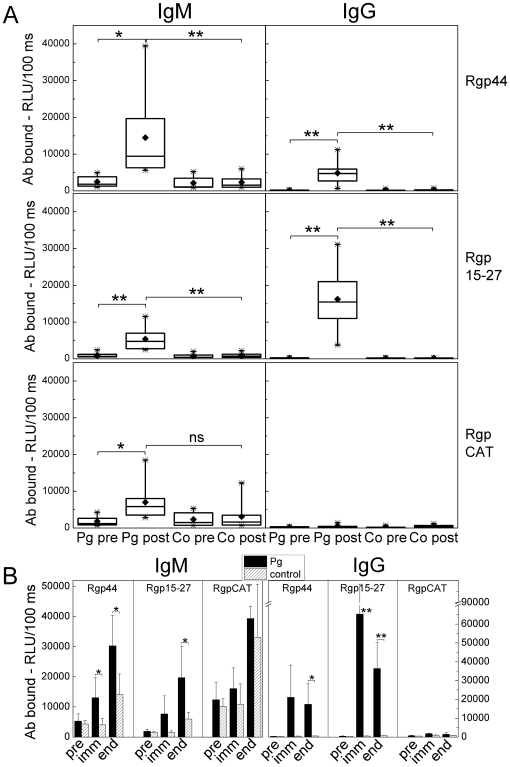
Antibodies to recombinant gingipain in *P. gingivalis* immunized mice. Recombinant proteins of the arginine specific gingipain protease of *P. gingivalis* were produced in *E.coli*: two proteins in the hemagglutinin/adhesion domain, Rgp44 and Rgp15–27, and the catalytic domain, RgpCAT, which were used in chemiluminescence immunoassay to determine mouse plasma (1∶500) IgM and IgG binding in *P. gingivalis* immunized (Pg) and control (Co) groups in both A) C57BL/6 and B) LDLR^−/−^ mice. A) For C57BL/6 immunized and control mice the samples (n = 8 each) were determined as duplicate before (pre) and after (post) immunization. Box-plots represent the distribution of the means of the sample duplicates. B) Two plasma samples within the group were pooled for immunized (black bars) and control (hatched bars) LDLR^−/−^ mice and measured in duplicate. Samples were collected before (pre), after the second booster immunization (imm) and at the end of HFD (end). Columns represent the mean ± SD of pooled samples in each group. **P<0.01 and *P<0.05.

Domains of an arginine specific gingipain protease were produced in *E. coli*, and mouse plasma IgM and IgG binding to two recombinant proteins in the hemagglutinin/adhesion domain, Rgp44 and Rgp15–27, and to recombinant catalytic domain, RgpCAT, were analyzed with chemiluminescence immunoassay in *P. gingivalis* immunized and control groups in both C57BL/6 (Fig. 4A) and LDLR^−/−^ mice (Fig. 4B). *P. gingivalis* immunization increased significantly the IgM binding to Rgp44 and Rgp15–27 compared to controls both in C57BL/6 (Fig. 4A) and in LDLR^−/−^ mice (Fig. 4B). After *P. gingivalis* immunization the difference between IgM binding to RgpCAT was not significant when comparing immunized C57BL/6 (Fig. 4A) or LDLR^−/−^ (Fig. 4B) mice to controls. *P. gingivalis* immunization increased also IgG binding to Rgp44 and Rgp15–27 in both mouse strains (Fig. 4A, B), but IgG responses to RgpCAT seemed to be absent.

### Plasma dilution curves of immunized mice

**Figure 5:**
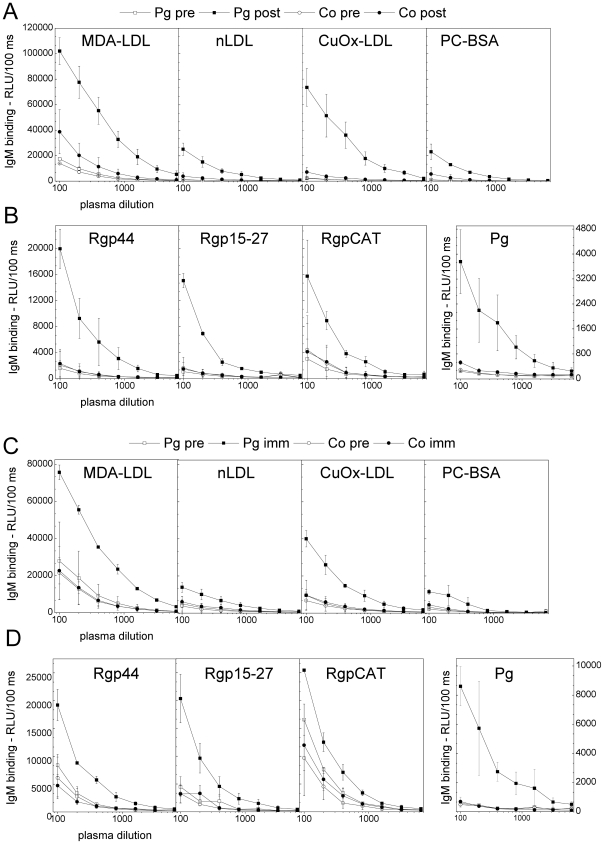
Dilution curves of mouse plasma IgM binding to antigens. A, B) C57BL/6 mice were immunized with heat-killed *P. gingivalis* ATCC33277 (Pg) and controls (Co) received saline. The plasma was diluted 1∶100–1∶6400 and IgM binding to MDA-LDL, CuOx-LDL, native LDL, PC-BSA (A), *P. gingivalis* and recombinant gingipain domains Rgp44, Rgp15–27 and RgpCAT (B) were determined before (pre) and after (post) immunization with chemiluminescence immunoassay. Mean ± SD for two samples is shown. C, D) LDLR^−/−^ mice were immunized with killed *P. gingivalis* (3 strains mixed) (Pg) and controls (Co) received PBS. Pooled plasma from two mice was used for each dilution curve, and mean ± SD for two dilution curves is shown. IgM binding to MDA-LDL, CuOx-LDL, native LDL, PC-BSA (C), *P. gingivalis* and recombinant gingipain domain Rgp44, Rgp15–27 and RgpCAT (D) were determined before (pre) and after the second booster immunization (imm) as described.

Dilution curves of plasma IgM binding to MDA-LDL, CuOx-LDL, native LDL and PC-BSA are shown in Figure 5 (A, C) for C57BL/6 and LDLR^−/−^ mice. No IgM binding to PC-BSA was demonstrated on dilution curves of Pg-immunized mouse plasma of C57BL/6 and LDLR^−/−^ mice (Fig. 5A, C). Dilution curves of plasma IgM binding to *P. gingivalis* and recombinant gingipain domains Rgp44, Rgp15–27 and RgpCAT are shown in Figure 5B for C57BL/6 mice and in Figure 5D for LDLR^−/−^ mice.

### Immunization with *P. gingivalis* increases IgM binding to apoptotic cells

**Figure 6:**
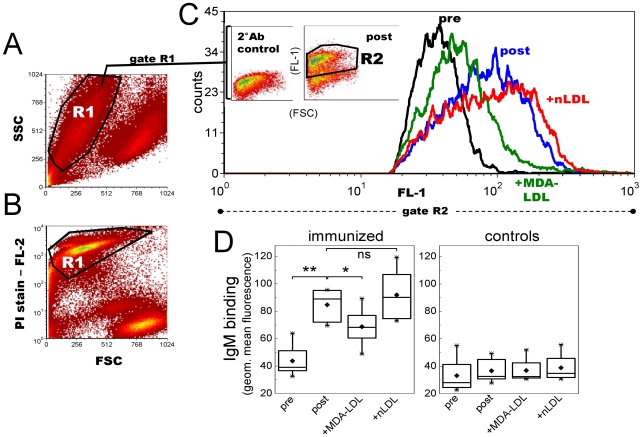
Mouse plasma IgM binding to apoptotic T lymphocytes after *P. gingivalis* immunization. C57BL/6 mice were immunized with heat-killed Pg and controls received sterile saline (n = 8 per group). Mouse plasma (1∶70) IgM binding to UV-irradiated Jurkat T cells was measured with flow cytometry. A, B) Apoptotic T cell population (R1) was verified with propidium iodide (PI) staining. C) Plasma IgM binding in gate R2 of preimmune (black) and postimmune (blue) plasma samples, and competition of IgM binding with 250 µg/ml MDA-LDL (green) or native LDL (red). Inset plots (in Fig. 6C) represent the secondary antibody control (2°Ab control) and plasma IgM binding to apoptotic cells in a Pg-immunized mouse (post). D) IgM binding to Jurkat cells was determined for each mouse in Pg-immunized and control group as geometric mean value in R2 subtracted by the 2°Ab control. Box-plot graphs represent the distribution of sample means calculated for two repeated assays. **P<0.01 and *P<0.05.

Plasma samples of the Pg-immunized and control C57BL/6 mice before and after immunization were analyzed for IgM binding to DAMPs on UV-irradiated apoptotic Jurkat T cells by flow cytometry (Fig. 6A, B). Pg-immunized mice had significantly elevated plasma IgM binding to apoptotic cells (Fig. 6C, D) whereas the controls did not show any increase (Fig. 6D). Induction of MDA-LDL specific antibodies in response to bacterial immunization was further verified by competitive incubation of immune plasma with MDA-modified or native LDL. MDA-LDL, but not native LDL, competed for IgM binding to apoptotic cells in Pg-immunized mice resulting in approximately 20–30% reduction in IgM binding (Fig. 6C, D). Controls showed no difference in the competitive flow cytometry assay (Fig. 6D).

### *P. gingivalis* immunization reduces lipid deposition in the aorta of LDLR^−/−^ mice

**Figure 7:**
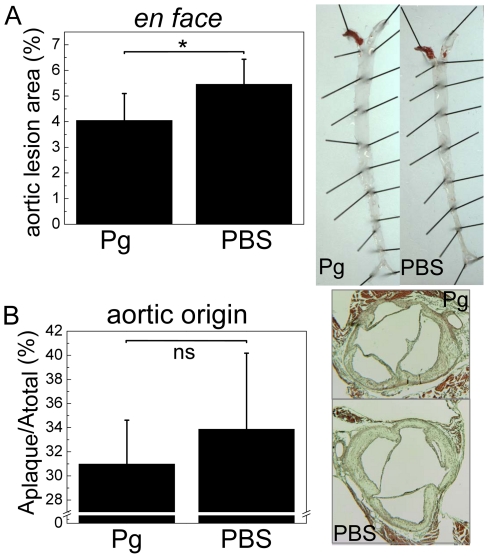
Quantification of atherosclerosis in LDLR^−/−^ mice immunized with *P. gingivalis*. LDLR^−/−^ mice (n = 7) were immunized without adjuvant with killed *P. gingivalis* (3 strains mixed) (Pg) followed by high fat diet (HFD). Controls (PBS, n = 8) received PBS. A) The extent of atherosclerotic plaque development was determined after HFD by *en face* analysis of the Sudan IV -stained aortas. B) Lesions at the aortic origin were measured on histological sections as percentage of plaque area in the aorta cross-sectional area. Representative pictures of aortas and cross-sections are shown for each group. * P<0.05.

Atheroprotective effect of bacteria bearing MDA-LDL cross-reactive epitopes was tested by immunizing LDLR^−/−^ mice with killed *P. gingivalis*. Significantly smaller areas of lipid deposition were measured in Pg-immunized mice compared to controls by *en face* lipid staining analysis (Fig. 7A). Plaque sizes at the aortic origin of Pg-immunized mice did not differ significantly from the plaques sizes of the controls (Fig. 7B).

### Human serum contains IgM recognizing cross-reactive epitopes on MDA-LDL and *P. gingivalis*

**Figure 8:**
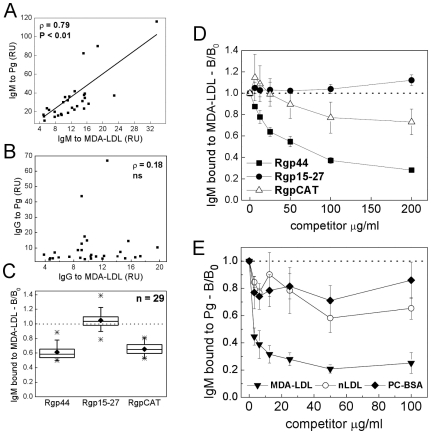
Association between human serum IgM to *P. gingivalis* and MDA-LDL, and competitive binding with recombinant gingipain domains. Sera from 29 healthy adults were analyzed for IgM (A) and IgG (B) binding to Pg and MDA-LDL by chemiluminescence immunoassay. Associations between antibody levels were analyzed with Spearman rank correlation test. Human sera were pre-incubated with recombinant gingipain domains Rgp44, Rgp15–27, RgpCAT (C, D) in a competitive immunoassay detecting IgM binding to immobilized MDA-LDL. The ratio of serum IgM binding (B/B_0_) to MDA-LDL with and without competitor (175 µg/ml) in 29 human serum samples (C) and dose-dependent competition assays of one sample (D). Reciprocal competition assay was performed to analyze human serum IgM binding to Pg antigen competed with MDA-LDL, nLDL and PC-BSA in a representative sample (E). RU, relative units.

The presence of antibodies binding to cross-reactive epitopes on OxLDL and *P. gingivalis* was assessed also in humans. Serum samples (n = 29) were analyzed for IgM binding to MDA-LDL, *P. gingivalis* and recombinant gingipain domains with chemiluminescence immunoassays. Serum IgM binding to MDA-LDL associated positively with IgM binding to *P. gingivalis* (Spearman ρ = 0.79, P<0.01) (Fig. 8A) but there was no association between serum IgG to *P. gingivalis* and MDA-LDL (Fig. 8B). Recombinant gingipain domains Rgp44 and RgpCAT competed effectively for serum IgM binding to MDA-LDL in a dose-dependent manner in competitive immunoassays (Fig. 8 C, D). Rgp15–27 did not compete for human serum IgM binding to MDA-LDL (Fig. 8C, D). Human serum IgM binding to *P. gingivalis* was effectively competed with MDA-LDL but not with native LDL or PC-BSA (Fig. 8E).

## Discussion

The present study demonstrated new epitope cross-reactivity between oxidized LDL and bacterial epitopes. Mouse monoclonal IgM specific for malondialdehyde modified LDL (MDmAb) recognized epitopes on the major periodontal pathogen *P. gingivalis*. Immunization of mice with killed *P. gingivalis* increased plasma IgM binding to MDA-LDL and DAMPs present on apoptotic cells. Pg-immunization attenuated development of atherosclerosis in LDLR^−/−^ mice, and we suggest that one possible mechanism contributing to the diminished aortic lipid deposition may have been the induction of IgM antibodies to MDA-LDL. *P. gingivalis* proteins recognized by MDmAb were identified using mass spectrometry, and target epitopes were located on gingipains, which are special proteases of *P. gingivalis* and crucial for its virulence. The positive association between human serum IgM binding to *P. gingivalis* and MDA-LDL, and the recognition of recombinant gingipain domains by serum IgM suggested relevance of these findings also in humans.

Natural IgM antibodies bind to oxidized lipid and protein epitopes generated on various molecules including LDL particle undergoing oxidation and ageing cells exposed to burst of oxidative stress [10]. At least two types of epitopes on oxidized phospholipids are previously reported to display molecular mimicry with epitopes on microbes. Many bacteria express epitopes containing PC, including approximately 30% of the strains colonizing the oral cavity such as *Aggregatibacter actinomycetemcomitans*, *Fusobacterium nucleatum* and *Streptococcus sanguis* [49], [50]. Bacterial strains can be classified as PC-bearing or devoid of PC according to their ability to incorporate choline from the culture medium [30]. A significant human pathogenic bacterium *Streptococcus pneumoniae* bears PC epitopes on its cell wall, and there is evidence that PC induces protective immune response in animal models of pneumococcal infection [51]. Natural IgMs in mice are reported to recognize the PC moiety on *S. pneumoniae* cell wall polysaccharide and on the oxidized phospholipids on LDL [7]. Association studies show an inverse relationship between anti-PC IgM and risk for CVD, however, human IgM antibodies recognizing bacterial PC epitopes have not been shown to play a definite role in atherogenesis [20]. Elevated anti-PC antibodies have been demonstrated in periodontitis patients compared to those with no attachment loss [30] and to those with less suppuration [52]. Studies also demonstrate recognition of PC moieties on oxidized LDL by serum and gingival crevicular fluid anti-PC antibodies [50], [53]. The other oxidized epitope displaying molecular mimicry with bacterial epitopes is cardiolipin, a phospholipid located in the inner leaflet of mitochondria and present also in OxLDL and bacteria. Cardiolipin is oxidized during apoptosis, and natural antibodies to oxidized cardiolipin bind to apoptotic cells and OxLDL [45]. IgG-antibodies to both PC [30] and cardiolipin [54] have been shown to associate with both atherosclerosis and periodontitis.

Bacterial strains devoid of PC are able to absorb anti-OxLDL antibodies from human sera [53] suggesting presence of additional cross-reactive epitopes in oral bacteria. Based on that, we determined to screen few bacterial species for recognition by anti-MDA-LDL IgM antibodies. Since some *P. gingivalis* strains have previously been determined as devoid of PC [30], we wanted to analyze the cross-reactivity of anti-OxLDL antibodies with bacterial epitopes other than PC. Natural IgMs binding to MDA epitopes were chosen for the study since MDA is the most abundant reactive aldehyde generated by lipid peroxidation in biological systems, and IgM antibodies binding to MDA-modified proteins have been described in humans [12], [17].

Apoptosis is known to generate DAMPs on cell membrane phospholipids and proteins due to disturbed integrity of the bilayer membrane and oxidative attack [55], [56]. Recognition of apoptotic cells containing oxidized lipids and proteins is a well-characterized feature of natural IgM antibodies [10], [18], [45], [55]. Monoclonal IgM selected for binding to either the PC moiety or the MDA epitopes on LDL bind to apoptotic, but not to viable cells, and in addition, these IgMs inhibit phagocytosis of apoptotic cells by macrophages [55]. To our knowledge no previous reports describe the increased IgM binding to apoptotic cells in mice following immunization with killed bacteria, such as the periodontal pathogen *P. gingivalis* in the present study. MDA-LDL competed effectively with the post-immunization plasma for the IgM binding to apoptotic cells yielding 20–30% reduction in IgM binding. This finding suggests that bacterial antigenic stimuli induced production of IgM that recognizes MDA-LDL through molecular mimicry. However, the rest of the induced IgM was presumably directed towards epitopes other than MDA, since whole cell bacterial antigen was used for immunizations, and therefore, it is likely that additional immune responses were generated against various other bacterial epitopes. Presumably many different bacteria contain epitopes that are recognized by natural IgMs binding to MDA epitopes, and thus, it can be hypothesized that IgM binding to apoptotic cells could also increase upon immunization with other bacteria, not only *P. gingivalis*.

The present study showed reduced lipid deposition in the aorta suggesting attenuation of atherogenesis after immunizing LDLR^−/−^ mice with killed *P. gingivalis*. This is in accordance with the previous studies [25], [26], [28] all performed with apoE -deficient mice that spontaneously develop lesions even on regular chow diet [57]. In this study we used LDLR^−/−^ mice which have no considerable lesion progression before starting the HFD [58]. We also immunized C57BL/6 mice with *P. gingivalis* and demonstrated the increased IgM binding to MDA-LDL even without atherogenic diet. The result suggests that epitopes on bacteria, for example gingipain of *P. gingivalis*, induce production of natural IgM capable of binding to OxLDL independently of excess cholesterol. C57BL/6 and LDLR^−/−^ mice immunized with killed *P. gingivalis* had a similar pattern of anti-MDA-LDL antibody production; IgM binding to MDA-LDL was increased but IgG antibodies with this specificity were not produced. We suggest that bacterial immunization induces anti-MDA-LDL IgM production, which could prevent the progression of atherosclerosis by diminishing uptake of OxLDL by macrophages and thereby reduce the formation of foam cells at sites of developing atherosclerotic lesions. Our data on Pg-immunizations also suggests that only innate IgM antibodies recognizing OxLDL were produced whereas IgG to OxLDL was absent.

The specificity of natural IgM to MDA epitopes induced after immunization with *P. gingivalis* was evaluated by additional measurements of IgM binding to PC-BSA and CuOx-LDL, which contains PC epitopes. Both C57BL/6 and LDLR^−/−^ mice immunized with *P. gingivalis* showed elevated IgM binding to CuOx-LDL but there was no substantial increase in IgM binding to PC when conjugated with a simple BSA carrier molecule. CuOx-LDL contains several types of oxidization-derived epitopes and MDA-epitopes have been reported [11] to be among them besides the major epitope type of phosphocholine.

The present study showed that epitopes for MDmAb binding on *P. gingivalis* are sequences in gingipain proteases. Gingipain amino acid sequence contains numerous repeated sequences [59] which are possibly explaining the observed differences in the recognition of the three recombinant Rgp domains by mouse and human IgM. Different exposure to antigens between humans and mice and polyclonal nature of serum IgM might additionally explain the different binding patterns of gingipain recognition. The different binding pattern of MDmAb to Rgp15–27 in Western blot and competitive liquid-phase immunoassays can be due to differences in the experimental conditions i.e. reducing and denaturing versus the native liquid-phase. Additionally, the different binding pattern can be due to conformational changes revealing cryptic epitope presentation similar to that described e.g. for autoantibodies binding to β2-glycoprotein 1 [60], [61].

The identified targets for MDmAb, the gingipain proteases, have been characterized in-depth [62], and interestingly, proposed as potential targets for protective vaccination against periodontitis [46], [63]. However, none of the previous work describes the binding of natural IgM to gingipain of *P. gingivalis*. Human and mouse monoclonal IgG antibodies binding to hemagglutinin domain of gingipain have been described and shown to inhibit hemagglutinating activity of *P. gingivalis* [64]. These studies [63], [64] demonstrate recognition of similar sized protein fragments as those identified by MDmAb of the present study. Epitope mapping of these IgGs has revealed peptide sequences (e.g. PVQNLT) that are present on gingipain hemagglutinin domain [64]. The true identity of the epitope on gingipain that is recognized by the MDA-specific IgM remains yet to be solved, and the question remains open whether the molecular mimicry between MDA-LDL and gingipain is only recognized by natural IgM or could anti-gingipain-IgG antibodies also play a role.

Intriguingly, the present data can be linked to numerous association studies establishing that periodontal infections increase risk for developing atherosclerosis [21], [65]. In more detail, high serum IgG titers to *P. gingivalis* have been shown to predict myocardial infarction [66], and additionally, antibody responses to other periodontal pathogens are associated with CVD [22]. The epitope cross-reactivity of MDA-LDL and *P. gingivalis* gingipain suggests recognition by similar types of natural IgM clones in humans and mice. Further, our hypothesis is that these types of natural IgM could confer protection against harmful agents associated with the development of periodontitis-induced atherosclerosis. To obtain further confirmation for the hypothesis, a highly relevant experiment would be an immunization of mice with recombinant Rgp44 cloned from the adhesion/hemagglutinin domain of *P. gingivalis* gingipain followed by the introduction of atherogenic diet. If the peptide sequence of Rgp44 could elicit elevated levels of IgM binding to MDA-LDL, the Rgp44-immunization could result in amelioration of atherosclerosis. Plasma IgM binding to MDA-LDL has been described in cohort studies on a population level and it has been associated with the risk for atherosclerosis [12], [67]. Thus, we conclusively suggest that alteration in each individual's natural antibody repertoire against MDA-modified oxidized epitopes could also affect recognition and elimination of periodontitis pathogens.

Molecular mimicry between epitopes on MDA-LDL and *P. gingivalis* gingipain protease was shown to be recognized by natural IgM of the innate immune system. Nevertheless, the role of the previously characterized cross-reactivity of the PC epitope between OxLDL and bacteria recognized by natural monoclonal antibodies [7], [31] other than the MDmAb of the present study should not be omitted. The present study broadens furthermore the notion of various PAMPs present on OxLDL and bacteria, and possibly offers new insights into resolving the relationship between periodontal infections and atherosclerosis.

## Supporting Information

Figure S1 **Protein identification for 45 kDa band 1.** **A**) Mascot score histogram. Individual ions scores >26 indicate identity or extensive homology (P<0.05), protein scores are derived from ions scores as a non-probabilistic basis for ranking protein hits. **B**) Gingipain amino acid sequence with the matching tryptic cleavage peptide sequence highlighted in red. **C**) MSMS spectrum showing the matching amino acids in the peptide sequence.
(PPT)

Figure S2 **Protein identification for 40 kDa band 2.** **A**) Mascot score histogram. Individual ions scores >26 indicate identity or extensive homology (P<0.05), protein scores are derived from ions scores as a non-probabilistic basis for ranking protein hits. Gingipain R2 (RgpB) was also identified as a protein matching the same set of peptides. (score 89, not shown). **B**) Gingipain amino acid sequence with the matching tryptic cleavage peptide sequence highlighted in red. **C**) MSMS spectrum showing the matching amino acids in the peptide sequence.
(PPT)

Figure S3 **Protein identification for 32 kDa band 3.** **A**) Mascot score histogram. Individual ions scores >26 indicate identity or extensive homology (P<0.05), protein scores are derived from ions scores as a non-probabilistic basis for ranking protein hits. **B**) Hemagglutinin A amino acid sequence with the matching tryptic cleavage peptide sequences highlighted in red. **C**) MSMS spectrum showing the matching amino acids in the peptide sequence.
(PPT)

Figure S4 **IgM binding to oxidized cardiolipin.** Cardiolipin (CL) in ethanol was purchased from Sigma Aldrich (St. Louis, MO, USA) and used 30 µg/ml in absolute ethanol for coating microtiter plates 25 µl/well during 20 min evaporation in a hood. Then CL was allowed to oxidize at room temperature for 3.5 hours before mouse plasma (1∶500) or monoclonal IgM (MDmAb or LRO1, 2.5 µg/ml) samples diluted in 0.5% fish gelatin were added [45]. Chemiluminescence immunoassay was continued as described in the article text. A) Mouse monoclonal IgM specific for MDA-LDL (MDmAb) or oxidized CL (LRO1) was tested for binding to *P. gingivalis* antigen representing either the strain ATCC33277 only or three strains (ATCC 33277, W50 and OMGS 434) mixed and absorbance of the solution was adjusted to 0.15 at 580 nm. B) IgM binding to CL in C57BL/6 mice immunized with *P. gingivalis* (Pg) and in saline immunized controls (Co) before (pre) and after immunization (post). For each mouse the background of IgM binding to ethanol coated plate was subtracted. RLU, relative light units.
(PDF)

Figure S5 **Antibodies to MDA-LDL before immunization of LDLR^−/−^ mice.** Plasma IgM and IgG binding to MDA-LDL was determined with chemiluminescence immunoassay in *P. gingivalis* (Pg, n = 7) or control immunized (Co, n = 8) LDLR^−/−^ mice. All plasma samples were diluted 1∶1000, and samples before immunization (pre) were analyzed for each mouse separately in duplicate. As an assay control demonstrating the relative differences in antibody binding the samples after second booster immunization (imm) and at the end of hig fat diet (end) were prepared for both groups (Pg and Co) by pooling leftover plasma samples between 2, 3 or 4 mice.
(PDF)

Methods S1 (DOC)
