# Supplementary material for: Recognition of Porphyromonas gingivalis Gingipain Epitopes by Natural IgM Binding to Malondialdehyde Modified Low-Density Lipoprotein
Source: PLoS One. 2012 Apr 5;7(4):e34910. doi: 10.1371/journal.pone.0034910 (PMC3320647; doi:10.1371/journal.pone.0034910)
Supplement: Figure S1 — Protein identification for 45 kDa band 1. A) Mascot score histogram. Individual ions scores >26 indicate identity or extensive homology (P<0.05), protein scores are derived from ions scores as a non-probabilistic basis for ranking protein hits. B) Gingipain amino acid sequence with the matching tryptic cleavage peptide sequence highlighted in red. C) MSMS spectrum showing the matching amino acids in the peptide sequence. (PPT) [file pone.0034910.s001.ppt]

## Slide 1
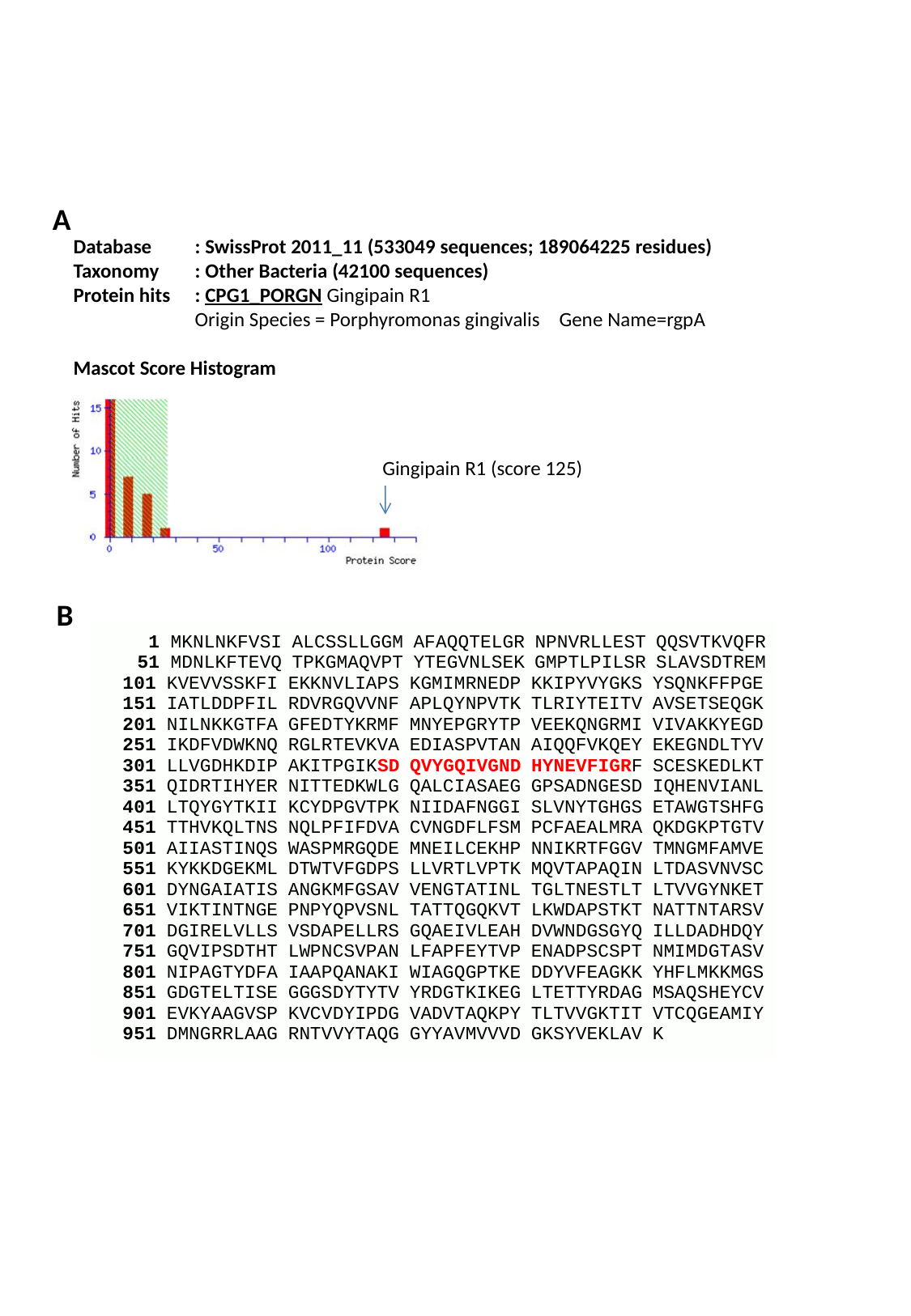

A
Database 	: SwissProt 2011_11 (533049 sequences; 189064225 residues)
Taxonomy 	: Other Bacteria (42100 sequences)
Protein hits 	: CPG1_PORGN Gingipain R1
	Origin Species = Porphyromonas gingivalis 	Gene Name=rgpA
Mascot Score Histogram
Gingipain R1 (score 125)
B

## Slide 2
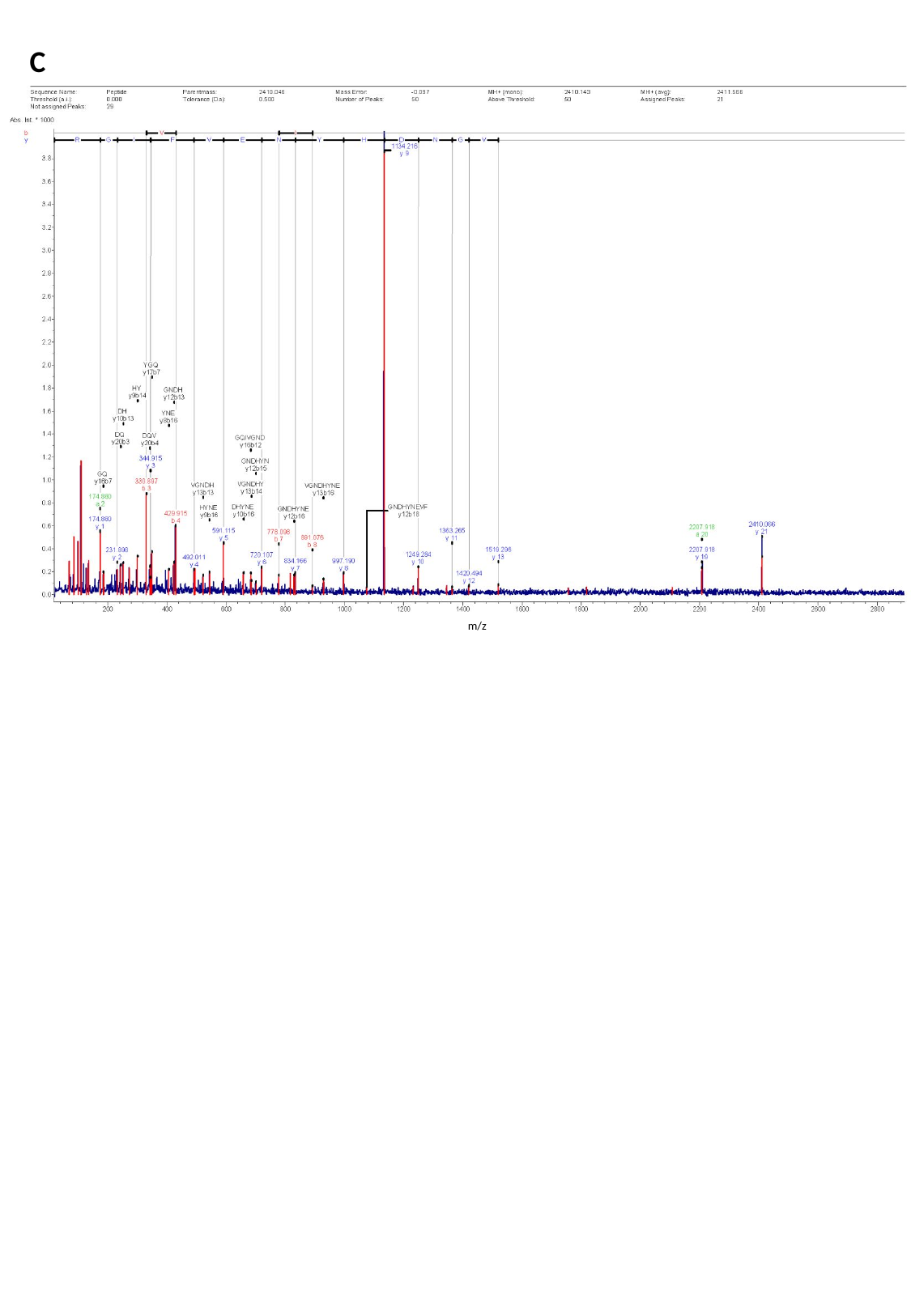

C
m/z
