# Supplementary material for: Recognition of Porphyromonas gingivalis Gingipain Epitopes by Natural IgM Binding to Malondialdehyde Modified Low-Density Lipoprotein
Source: PLoS One. 2012 Apr 5;7(4):e34910. doi: 10.1371/journal.pone.0034910 (PMC3320647; doi:10.1371/journal.pone.0034910)
Supplement: Figure S2 — Protein identification for 40 kDa band 2. A) Mascot score histogram. Individual ions scores >26 indicate identity or extensive homology (P<0.05), protein scores are derived from ions scores as a non-probabilistic basis for ranking protein hits. Gingipain R2 (RgpB) was also identified as a protein matching the same set of peptides. (score 89, not shown). B) Gingipain amino acid sequence with the matching tryptic cleavage peptide sequence highlighted in red. C) MSMS spectrum showing the matching amino acids in the peptide sequence. (PPT) [file pone.0034910.s002.ppt]

## Slide 1
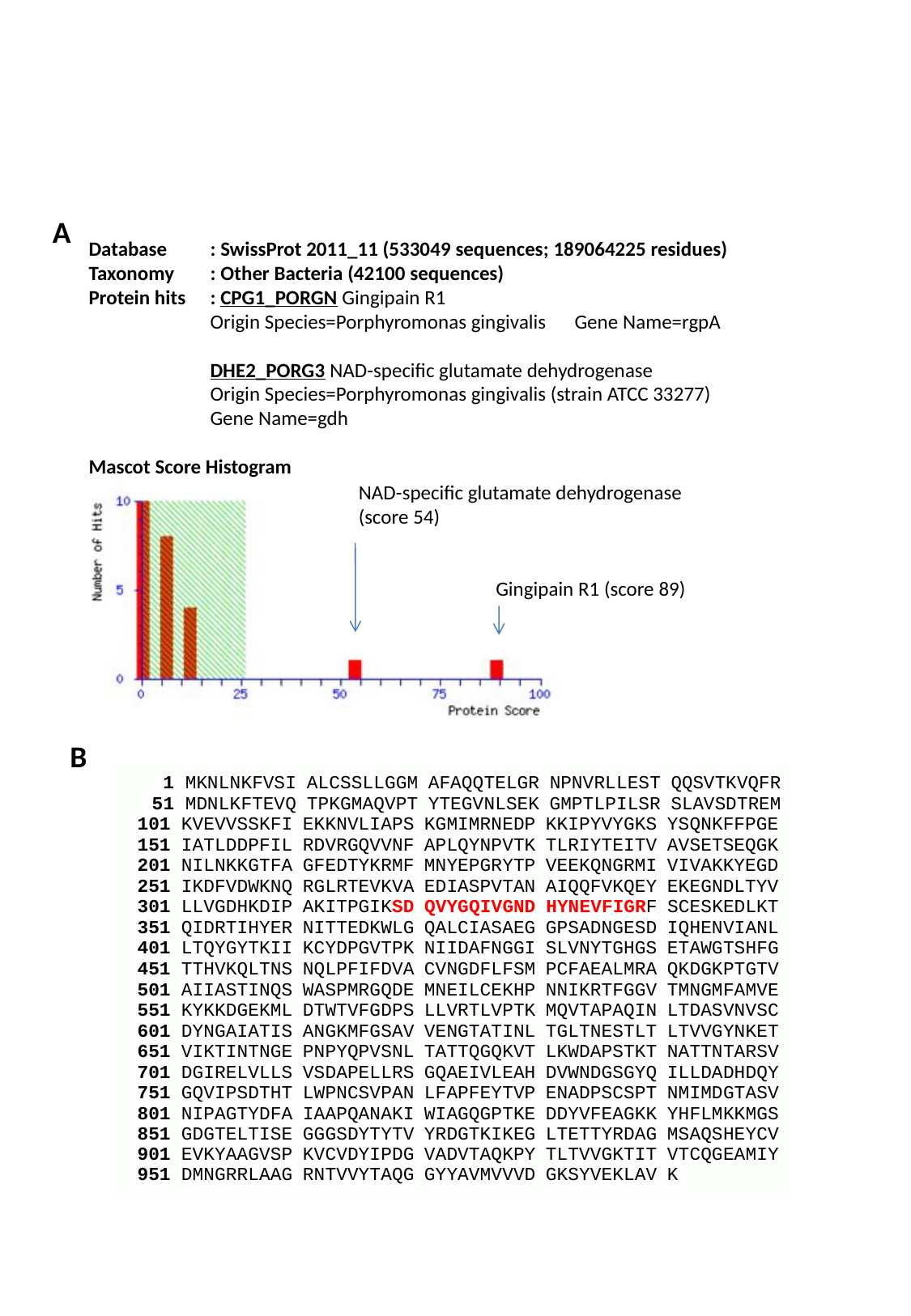

A
Database	: SwissProt 2011_11 (533049 sequences; 189064225 residues)
Taxonomy	: Other Bacteria (42100 sequences)
Protein hits	: CPG1_PORGN Gingipain R1
	Origin Species=Porphyromonas gingivalis 	Gene Name=rgpA
	DHE2_PORG3 NAD-specific glutamate dehydrogenase
	Origin Species=Porphyromonas gingivalis (strain ATCC 33277)
	Gene Name=gdh
Mascot Score Histogram
NAD-specific glutamate dehydrogenase
(score 54)
Gingipain R1 (score 89)
B

## Slide 2
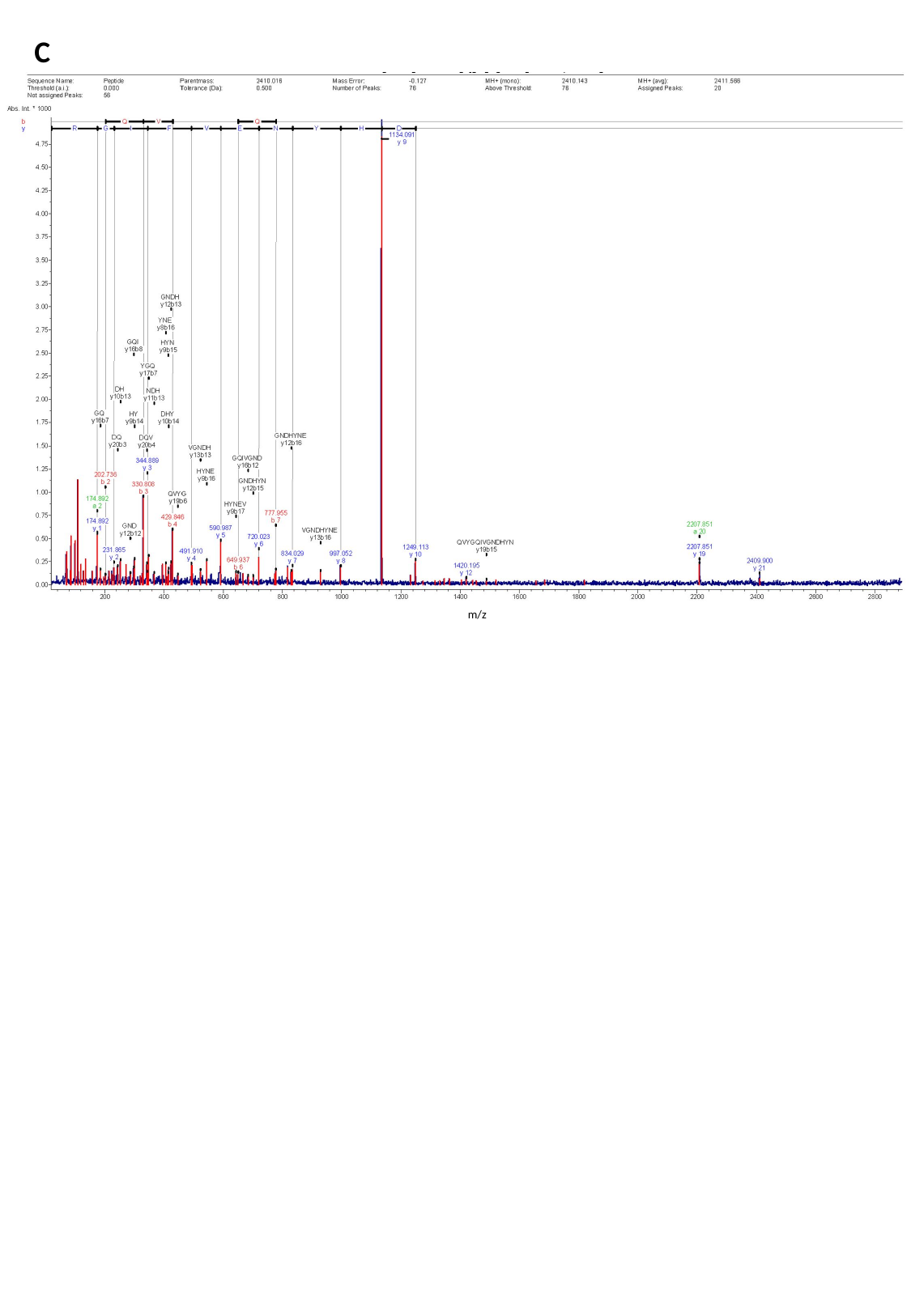

C
m/z
