# Supplementary material for: Recognition of Porphyromonas gingivalis Gingipain Epitopes by Natural IgM Binding to Malondialdehyde Modified Low-Density Lipoprotein
Source: PLoS One. 2012 Apr 5;7(4):e34910. doi: 10.1371/journal.pone.0034910 (PMC3320647; doi:10.1371/journal.pone.0034910)
Supplement: Figure S3 — Protein identification for 32 kDa band 3. A) Mascot score histogram. Individual ions scores >26 indicate identity or extensive homology (P<0.05), protein scores are derived from ions scores as a non-probabilistic basis for ranking protein hits. B) Hemagglutinin A amino acid sequence with the matching tryptic cleavage peptide sequences highlighted in red. C) MSMS spectrum showing the matching amino acids in the peptide sequence. (PPT) [file pone.0034910.s003.ppt]

## Slide 1
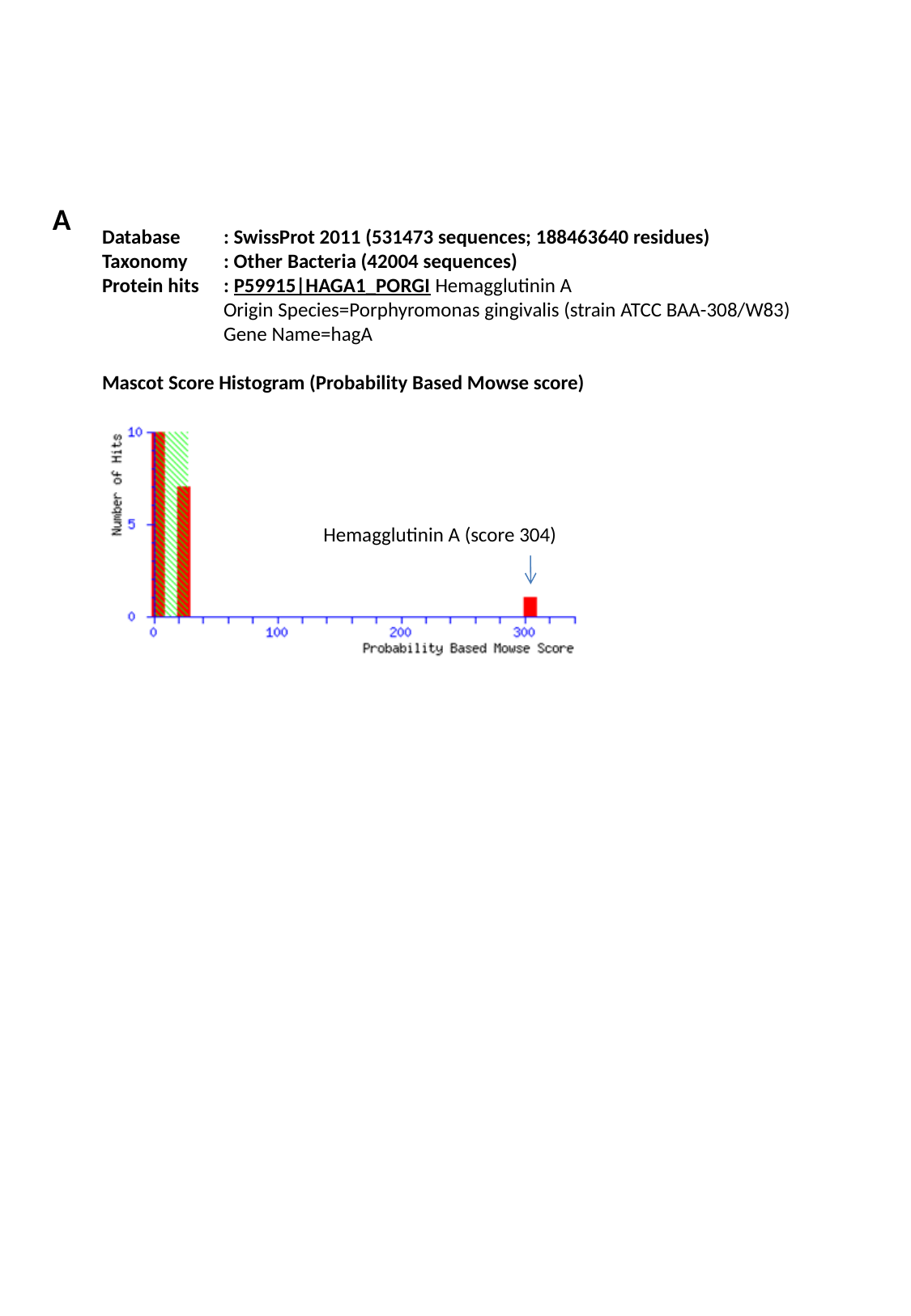

A
Database	: SwissProt 2011 (531473 sequences; 188463640 residues)
Taxonomy	: Other Bacteria (42004 sequences)
Protein hits	: P59915|HAGA1_PORGI Hemagglutinin A
	Origin Species=Porphyromonas gingivalis (strain ATCC BAA-308/W83)
	Gene Name=hagA
Mascot Score Histogram (Probability Based Mowse score)
Hemagglutinin A (score 304)

## Slide 2
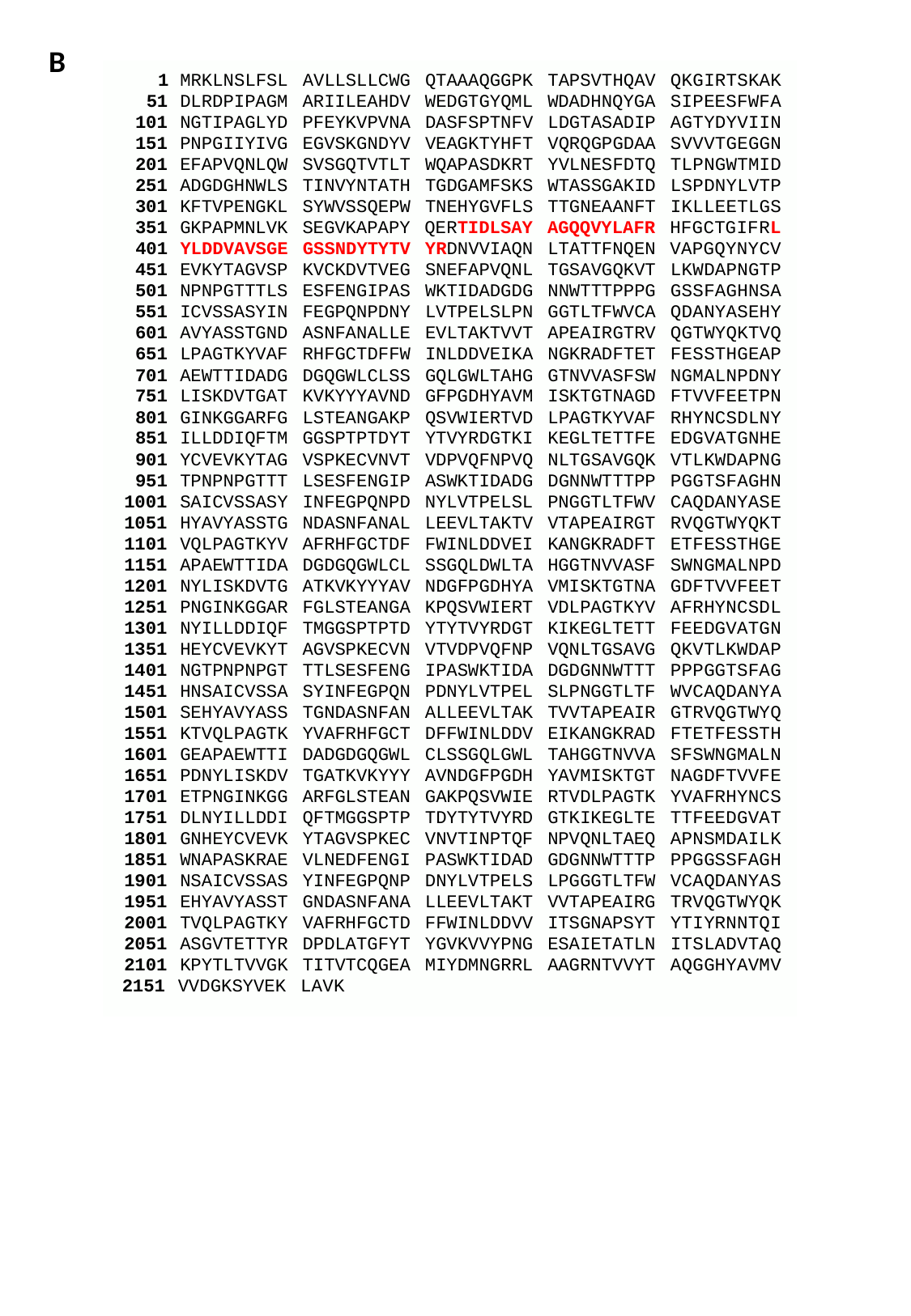

B

## Slide 3
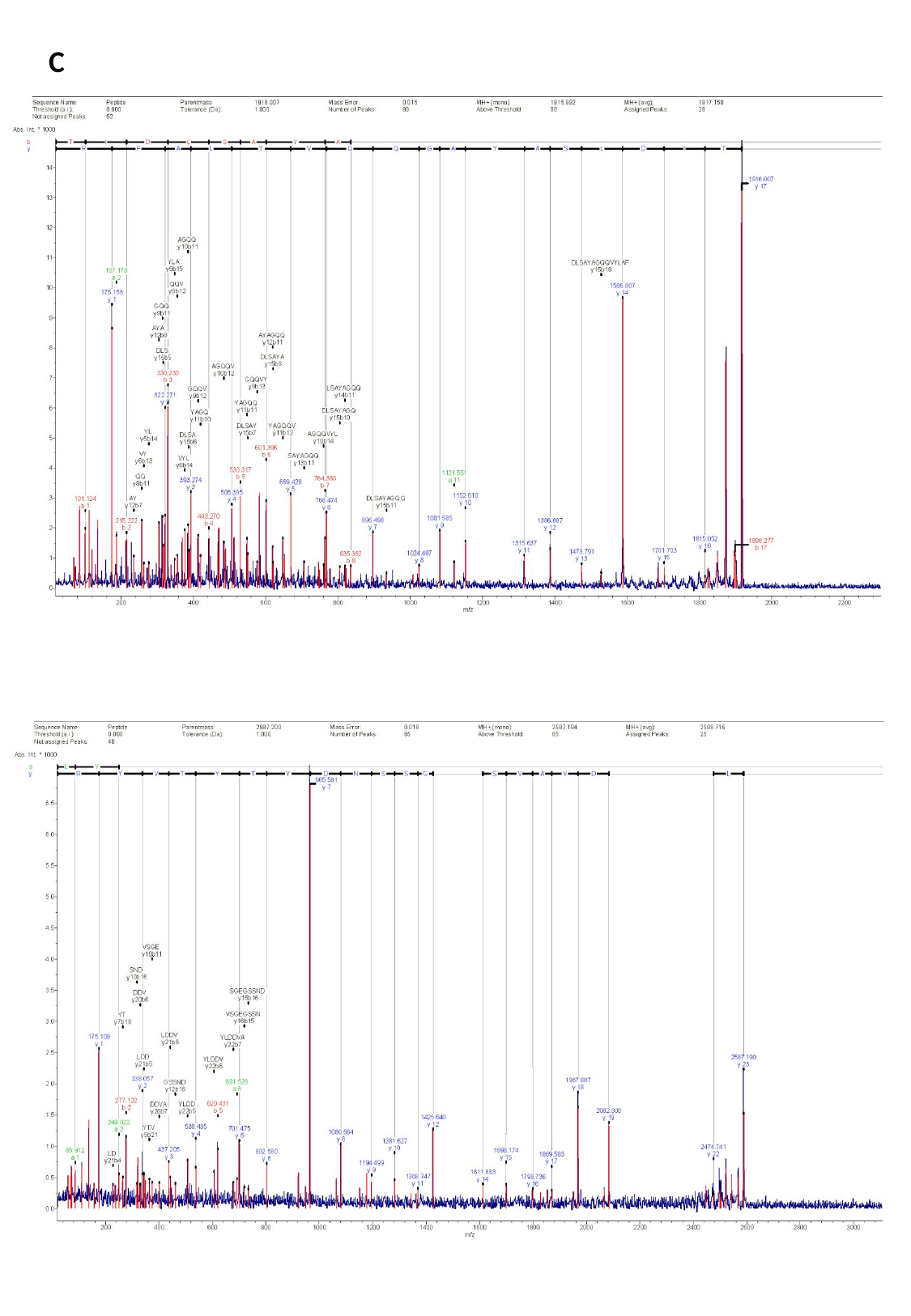

C
