# Supplementary figures and images for: Recognition of Porphyromonas gingivalis Gingipain Epitopes by Natural IgM Binding to Malondialdehyde Modified Low-Density Lipoprotein
Source: PLoS One. 2012 Apr 5;7(4):e34910. doi: 10.1371/journal.pone.0034910 (PMC3320647; doi:10.1371/journal.pone.0034910)

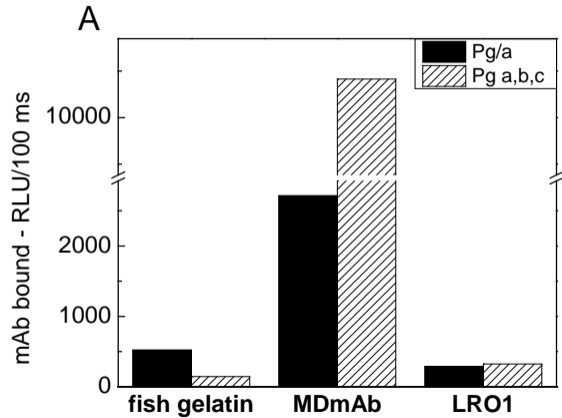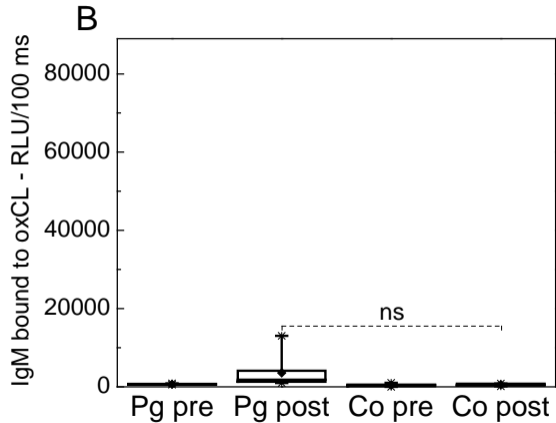

Supplement: Figure S4 — IgM binding to oxidized cardiolipin. Cardiolipin (CL) in ethanol was purchased from Sigma Aldrich (St. Louis, MO, USA) and used 30 µg/ml in absolute ethanol for coating microtiter plates 25 µl/well during 20 min evaporation in a hood. Then CL was allowed to oxidize at room temperature for 3.5 hours before mouse plasma (1∶500) or monoclonal IgM (MDmAb or LRO1, 2.5 µg/ml) samples diluted in 0.5% fish gelatin were added [45]. Chemiluminescence immunoassay was continued as described in the article text. A) Mouse monoclonal IgM specific for MDA-LDL (MDmAb) or oxidized CL (LRO1) was tested for binding to P. gingivalis antigen representing either the strain ATCC33277 only or three strains (ATCC 33277, W50 and OMGS 434) mixed and absorbance of the solution was adjusted to 0.15 at 580 nm. B) IgM binding to CL in C57BL/6 mice immunized with P. gingivalis (Pg) and in saline immunized controls (Co) before (pre) and after immunization (post). For each mouse the background of IgM binding to ethanol coated plate was subtracted. RLU, relative light units. (PDF) [file pone.0034910.s004.pdf]

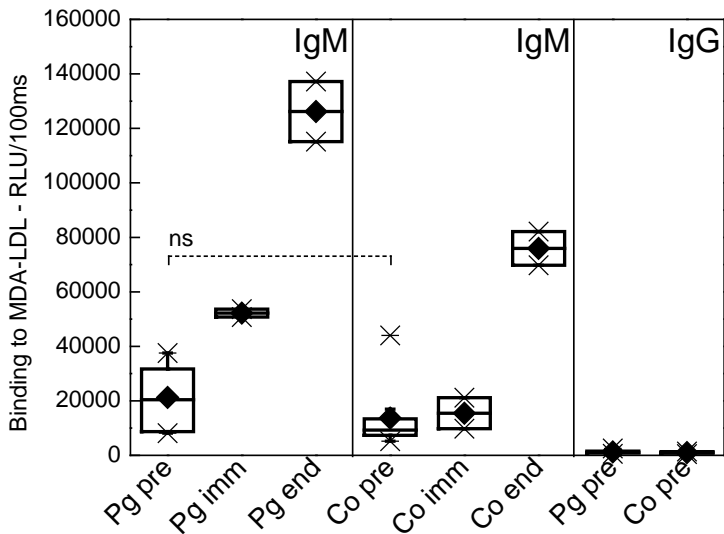

Supplement: Figure S5 — Antibodies to MDA-LDL before immunization of LDLR−/− mice. Plasma IgM and IgG binding to MDA-LDL was determined with chemiluminescence immunoassay in P. gingivalis (Pg, n = 7) or control immunized (Co, n = 8) LDLR−/− mice. All plasma samples were diluted 1∶1000, and samples before immunization (pre) were analyzed for each mouse separately in duplicate. As an assay control demonstrating the relative differences in antibody binding the samples after second booster immunization (imm) and at the end of hig fat diet (end) were prepared for both groups (Pg and Co) by pooling leftover plasma samples between 2, 3 or 4 mice. (PDF) [file pone.0034910.s005.pdf]
