# Supplementary material for: Recognition of Porphyromonas gingivalis Gingipain Epitopes by Natural IgM Binding to Malondialdehyde Modified Low-Density Lipoprotein
Source: PLoS One. 2012 Apr 5;7(4):e34910. doi: 10.1371/journal.pone.0034910 (PMC3320647; doi:10.1371/journal.pone.0034910)
Supplement: Methods S1 — (DOC) [file pone.0034910.s006.doc]

**Purification of recombinant gingipains**

Purification of the histidine-tagged recombinant proteins followed the protocols of ProBond Purification System (Invitrogen, Carlsbad, CA, USA) with slight modifications. *E. coli* BL21(DE3) were harvested by centrifugation at 3,000 × g for 10 minutes and suspended in native lysis buffer containing 50 mM Tris – 25 mM sodium chloride – 2.5 mM magnesium chloride – 0.1 mM calcium chloride pH 8.0. Lysozyme was added 1 mg/ml and incubated at 30 °C for 30 minutes. Bacteria were sonicated 7 × 10 seconds and treated with DNase I (Fermentas) 4 U/ml for 15 minutes on ice. Cellular debris was collected by centrifugation at 3,000 × g for 15 minutes. Soluble Rgp15-27 protein was purified from the supernatant. Rgp15-27 was soluble in native lysis buffer but before incubation with nickel chelating slurry the salt concentration of the sample was increased to reduce non-specific binding. This was achieved by adding 1/5 volume of 5 × purification buffer (250 mM NaH_2_PO_4_ pH 8.0 with 2.5 M sodium chloride) to yield 0.5M final concentration of sodium chloride before binding to slurry. Nickel chelating slurry was equilibrated with native binding buffer (50 mM NaH_2_PO_4_ pH 8.0 with 0.5 M sodium chloride) before adding Rgp15-27 sample for 1 hour incubation at room temperature in a roller. The slurry was centrifuged 800 × g for 1 minute and washed four times with aliquots of wash buffer (50 mM NaH_2_PO_4_ pH 8.0 - 0.5 M sodium chloride – 20 mM imidazole). Nickel chelating slurry with bound recombinant protein was transferred to a column and Rgp15-27 was eluted with elution buffer (50 mM NaH_2_PO_4_ pH 8.0 – 0.5 M sodium chloride – 250 mM imidazole) by collecting 2 ml fractions, which were analyzed for protein content with Lowry protein assay and SDS-PAGE. Major elution fractions containing most of the Rgp15-27 were combined.

Recombinant Rgp44 and RgpCAT were expressed as inclusion bodies remaining in the cell pellet after native lysis. Rgp44 and -CAT were purified from *E. coli* cells by denaturing lysis buffer (6 M guanidium hydrochloride – 20 mM sodium phosphate pH 7.8 – 0.5 M sodium chloride) for 10 minutes. The lysate was homogenized with a needle and a syringe and sonicated 3 × 5 seconds. Viscous cell debris was separated by ultracentrifugation at 100,000 × g for 30 minutes. Proteins solubilized into the supernatant by denaturation were bound to nickel chelating slurry in denaturing lysis buffer for 30 minutes incubation at room temperature in a roller. Phosphate buffers of native purification protocol without urea were used in subsequent wash and elution steps for Rgp44 and RgpCAT also. Purity of the histidine-tagged recombinant proteins was analyzed on SDS-PAGE. Small molecular weight protein contaminants were removed with Amicon Ultra-4 Ultracel MWCO 30,000 filters (Millipore) for immunoassays using Rgps as immobilized antigens.
